# Prostate-specific Membrane Antigen Based Antibody-drug Conjugates for Metastatic Castration-resistance Prostate Cancer

**DOI:** 10.7759/cureus.7147

**Published:** 2020-02-29

**Authors:** Muhammad O Niaz, Michael Sun, Marigdalia K Ramirez-Fort, Muhammad J Niaz

**Affiliations:** 1 Internal Medicine, Sharif Medical City Hospital, Lahore, PAK; 2 Internal Medicine, Weill Cornell Medicine, New York, USA; 3 Life Sciences, Biofort Corp., Guaynabo, PRI; 4 Urology, Weill Cornell Medicine, New York, USA; 5 Physiology / Pathology, San Juan Bautista School of Medicine, Caguas, PRI

**Keywords:** prostate cancer, prostate-specific membrane antigen, monoclonal antibody, antibody-drug conjugates

## Abstract

Cancer cells can be selectively targeted by identifying and developing antibodies to specific antigens present on the cancer cell surface. Cytotoxic agents can be conjugated to these antibodies that bind to these cell surface antigens in order to significantly increase the therapeutic index of whichever cytotoxic agent is utilized. This approach of conjugating the cytotoxic drugs to antibodies to target specific surface antigens enhances the anti-tumor activity of antibodies and improves the tumor-to-normal tissue selectivity of chemotherapy. Critical parameters in the development of these antibody-drug conjugates include: 1) selection of most appropriate antigen, 2) the ability of an antibody to be internalized after binding to the antigen, 3) cytotoxic drug potency and 4) stability of the antibody-drug conjugate. For prostate cancer, prostate-specific membrane antigen (PSMA, also known as folate hydrolase-1) is the most validated theragnostic target to date. PSMA is overexpressed on the prostate cancer cell surface, which makes it an even better target for selective drug delivery through conjugated antibodies. Here, we review the PSMA-based antibody-drug conjugates for metastatic castration-resistance prostate cancer (mCRPC).

## Introduction and background

Prostate-specific membrane antigen (PSMA, also known as folate hydrolase-1 and glutamate carboxypeptidase II) is an integral non-shed membrane glycoprotein, which is highly expressed in prostate cancer but has limited expression in benign prostate and other normal non-prostatic tissues [1-3]. It is also expressed abundantly in the neo-vasculature of other solid tumors, but interestingly, it is not expressed by tumor-adjacent normal blood vessels [4-10]. Androgen blockade and deprivation increases PSMA expression [11]. It also has a unique property of getting internalized, once bound to a PSMA specific antibody [12]. All of the above-mentioned characteristics suggest PSMA as an ideal target for radio-ligand therapy as well as cytotoxic antibody-drug conjugates. So far, two PSMA-targeted antibody-drug conjugates have undergone clinical investigation using maytansinoid and auristatin drugs, MLN2704 and PSMA-Antibody Drug Conjugate (ADC), respectively. Herein, we summarize the PSMA-based antibody-drug conjugates clinical trials in the treatment of metastatic castration-resistance prostate cancer (mCRPC). Table 1 shows the salient characteristics of all clinical trials discussed in this review.

**Table 1:** Summary of all currently published PSMA-based ADC clinical trials PSMA: Prostate-specific membrane antigen; ADC: Antibody drug conjugate.

PSMA-based antibody-drug conjugates						
Antibody drug conjugate	Drug type	Antibody/target	Cancer	Developer	Study phase	NCT Number
MLN2704	Maytansinoid DM1	hJ591/PSMA	Prostate	Millennium Pharmaceuticals	Phase I and I/II	NCT00052000 and NCT00070837
PSMA-ADC	Monomethyl auristatin E (MMAE)	anti-PSMA fully human IgG1/PSMA	Prostate	Progenics/Seattle Genetics	Phase I and II	NCT01414283 and NCT01695044

## Review

Phase I trial of MLN2704 for mCRPC

MLN2704 is an immunoconjugate between maytansinoid-1 (DM 1) and the humanized J591 antibody (MLN591), and it is designed to deliver the maytansinoid antimicrotubule agent, DM 1, directly to prostate-specific membrane antigen (PSMA) expressing cells. J591 (MLN591) is an anti-PSMA monoclonal antibody, and it has the property of becoming internalized once bound to the extracellular domain of PMSA [12, 13]. A phase I study of PSMA-based antibody-drug conjugate, MLN2704, was conducted by Galsky et al. [14]. The primary objectives of the study were to determine a safe dose of MLN2704, establish pharmacokinetics of measurable components of MLN2704 after a single administration, define the immunogenicity of MLN2704, and obtain preliminary evidence of anti-tumor activity in patients with progressive metastatic castration-resistant prostate cancer (mCRPC). Patients with histologically confirmed progressive metastatic castration-resistant prostate cancer were eligible. Baseline imaging with CT, MRI, and bone scan was done to assess the tumor burden and later treatment response. One patient was treated initially with 18 mg/m^2^ of MLN2704. Successive single-patient cohorts were treated at the next higher dose level, after a three-week observation period. Ten dose levels were planned with increments of 80%, 60%, 40%, 30%, 30%, 30%, 30%, 30%, and 30% greater than the previous dose. Any grade 2 or more toxicity at the first five dose levels mandated cohort expansion and adoption of a more conservative dose-escalation scheme. Otherwise, at dose level six and above, three patients per cohort were enrolled in a traditional 3+3 escalation scheme. A total of 23 patients received MLN2704 at doses ranging from 18 to 343 mg/m^2^. Eighteen of these patients received more than three doses at four-week intervals. Two (22%) of the nine patients treated at 264 or 343 mg/m^2^ had more than 50% decrease in prostate-specific antigen (PSA), accompanied by measurable tumor regression in the patient treated at 264 mg/m^2^. Only one patient had dose-limiting toxicity (DLT) at a dose level of 343 mg/m^2^ (febrile neutropenia). Nausea, fatigue, and diarrhea were the most commonly encountered adverse effects during this study. Eight of 23 patients developed low-grade neuropathy, which appeared more frequently at higher doses. The maximum tolerated dose was not pursued because of the clinical activity and tolerability of MLN2704 at the dose levels already explored and the dose-dependent peripheral neuropathy. This phase I study demonstrated that antibody-drug conjugates use for metastatic prostate cancer is feasible and safe and it can open new doors for approaching prostate cancer treatment.

Phase I/II trial of MLN2704 for mCRPC

After a phase I study of MLN2704 for mCRPC demonstrated safety and anti-tumor effects, a phase I/II trial was planned [15]. The primary endpoint was a sustained PSA response of 50% or more, without evidence of disease progression. Anti-tumor activity, toxicity, and immunogenicity were assessed. Sixty-two patients with histologically confirmed mCRPC received MLN2704 at ascending doses on four schedules: 1) weekly, 2) every two weeks, 3) every three weeks, and 4) on days 1 and 15 of a six-week schedule with doses ranging from 60-426 mg/m^2^. On a weekly dosing schedule, three patients were treated at 60, 84, 118, and 165 mg/m^2^ doses each, with a total of 12 patients. At two weeks’ dosing schedule, three patients were treated at 120, 168, 236, while six patients were treated at 330 mg/m^2^, with a total 15 patients getting treated at this dosing schedule. At every three weeks dosing schedule, 14 patients were treated at 330 mg/m^2^, and four were treated at 426 mg/m^2^, with a total of 18 patients getting treated at this dosing schedule. On a six-week schedule with dosing on day 1 and day 15 followed by four weeks of no dose, a total of 17 patients were treated at 330 mg/m^2^. Neurotoxicity proved to be the dose-limiting toxicity (DLT), with 44 patients (71%) developing peripheral neuropathy, of which six (10%) had grade 3/4. Other common toxicities included nausea (61%), fatigue (60%), anorexia (39%), diarrhea (39%), constipation (34%), and AST/ALT elevation (19%). Overall efficacy was also limited, with only five patients (8%) experiencing 50% or more PSA decline; five (8%) had PSA stabilization lasting 90 days or more. Only two of 35 patients on the three-week and six-week schedules achieved more than 50% PSA decline. Rapid deconjugation of DM1 from the MLN591 was thought to be responsible for peripheral neuropathy. Both free DM1 and free MLN591 antibody were detectable shortly after the catabolism of MLN2704. Due to the narrow therapeutic window, with significant percentage of patients (71%) developing peripheral neuropathy, the drug was discontinued following the conclusion of phase I/II studies.

Phase I trial of PSMA-ADC for mCRPC

More recently, another PSMA-based antibody drug-conjugate, PSMA-ADC, has undergone phase I and II trials [16, 17]. PSMA-ADC is a fully human IgG1 monoclonal antibody conjugated to the microtubule disrupting agent monomethyl auristatin E (MMAE), via a di-peptide linker (valine-citrulline). The di-peptide linker is designed to be stable in blood but is efficiently cleaved intracellularly following uptake of the ADC into PSMA-expressing prostate cancer cells [18, 19]. A phase I multicenter study of PSMA-ADC was designed to identify the maximum tolerated dose (MTD) and DLT in patients with progressive metastatic castration-resistance prostate cancer who had received prior taxane-based chemotherapy [16]. Patients with pathologically confirmed, progressive metastatic castration-resistance prostate cancer were eligible. A total of 52 subjects received PSMA-ADC at doses ranging from 0.4 to 2.8 mg/kg. Subjects in the higher dose groups had significant prior exposure to abiraterone, while one additional subject (2.5 mg/kg) had received enzalutamide prior to study entry. Treatment with 1.8 mg/kg and higher was associated with reductions in PSA and circulating tumor cells (CTCs). Overall, six patients showed radiographic response on bone scan and CT; all of them were treated at 18 mg/kg or higher dose level. Neutropenia and peripheral neuropathy were identified as important first-cycle and late DLT, respectively. The most common toxicities were fatigue (40%), neutropenia (33%), nausea (29%), anorexia (27%), transaminitis (25%), constipation (23%), and vomiting (19%). Overall, PSMA-ADC was well tolerated, and the dose of 2.5 mg/kg was determined to be the MTD.

Phase II trial of PSMA-ADC for mCRPC

After successful completion of phase I study, which showed that PSMA-ADC could be administered safely at 2.5 mg/kg with sufficient anti-tumor activity, a phase II trial was planned [17]. The primary objectives of the study were to determine the safety and anti-tumor activity (including PSA, CTCs, and radiographic response) of PSMA-ADC. In this phase II study, 119 patients with mCRPC, who progressed following abiraterone/enzalutamide, were enrolled. Out of these 119 patients, 83 had previous taxane therapy while 36 patients were chemotherapy-naive. Patients were administered 2.5 or 2.3 mg/kg PSMA-ADC intravenously, every three weeks for up to eight cycles. PSA declines of 30% or more and 50% or more were seen in 35 (30%) and 17 (14%) patients, respectively. CTC counts showed a decline of 50% or more in 93 (78%) patients and conversion from unfavorable to favorable counts in 77 (47%) at any time during the study. Thirty-one (26.05%) patients had a measurable disease as defined by the RECIST criteria [20]. Out of these 31 patients, four (12.9%) had a partial response, 19 (61.3%) stable disease, and eight (25.8%) patients progressed. Low neuroendocrine serum markers and high PSMA expression were associated with better efficacy. The most common treatment-related grade 3 adverse events were neutropenia, fatigue, electrolyte imbalance, anemia, and neuropathy. Grade 1-2 neuropathy occurred in 33 (40%) patients who have had prior taxane therapy and 18 (50%) patients who were chemotherapy-naive. Two patients out of 34, treated at 2.5 mg/kg, and one patient out of 85 treated at 2.3 mg/kg, died of sepsis. 2.3 mg/kg was better tolerated than 2.5 mg/kg. PSA declines and radiologic evidence of anti-tumor activity were seen in chemotherapy-naive as well as heavily pretreated patients. Again, deconjugation of MMAE was considered to be the etiology of the observed neurotoxicity.

## Conclusions

Antibody-drug conjugate, as a concept, is very promising for clinical investigation and remains an active area of research. Although MLN2704 clinical trials resulted in an unfavorable safety profile due to instability of the antibody-drug conjugate, it validated PSMA as an important immunoconjugate target. With a di-peptide linker, PSMA-ADC was associated with lower but still significant rates of neurotoxicity, again due to deconjugation. As novel and more effective linker agents are developed and enter clinical investigation, adverse events will decrease, while the efficacy of antibody-drug conjugates will improve.
